# Cultural bias in motor function patterns: Potential relevance for predictive, preventive, and personalized medicine

**DOI:** 10.1007/s13167-021-00236-3

**Published:** 2021-03-03

**Authors:** Karen Otte, Tobias Ellermeyer, Masahide Suzuki, Hanna M. Röhling, Ryota Kuroiwa, Graham Cooper, Sebastian Mansow-Model, Masahiro Mori, Hanna Zimmermann, Alexander U. Brandt, Friedemann Paul, Shigeki Hirano, Satoshi Kuwabara, Tanja Schmitz-Hübsch

**Affiliations:** 1 Experimental and Clinical Research Center, Charité - Universitätsmedizin Berlin Corporate Member of Freie Universität Berlin, Humboldt-Universität zu Berlin, and Berlin Institute of Health and Max Delbrück Center for Molecular Medicine, Lindenberger Weg 80, 113125 Berlin, Germany; 2 Motognosis GmbH, Berlin, Germany; 3 Department of Neurology, Vivantes Auguste-Viktoria-Klinikum, Berlin, Germany; 4 Department of Neurology, Graduate School of Medicine, Chiba University, Chiba, Japan; 5 NeuroCure Clinical Research Center, Charité - Universitätsmedizin Berlin, Corporate Member of Freie Universität Berlin, Humboldt-Universität zu Berlin, and Berlin Institute of Health, Berlin, Germany; 6 Division of Rehabilitation Medicine, Chiba University Hospital, Chiba, Japan; 7 Einstein Center for Neuroscience, Berlin, Germany; 8 Department of Experimental Neurology and Center for Stroke Research, Charité – Universitätsmedizin Berlin, Berlin, Germany; 9 Department of Neurology, University of California, Irvine, CA USA; 10 Department of Neurology, Charité – Universitätsmedizin Berlin, corporate member of Freie Universität Berlin, Humboldt-Universität zu Berlin, and Berlin Institute of Health, Berlin, Germany

**Keywords:** Personalized monitoring, Sub-optimal health, Motion capture, Motor biomarker, Gait analysis, Balance, Posturography, BMI, Neurodegenerative disorders, Risk assessment, Predictive preventive personalized medicine (PPPM/3PM), Cultural bias

## Abstract

**Background:**

Quantification of motor performance has a promising role in personalized medicine by diagnosing and monitoring, e.g. neurodegenerative diseases or health problems related to aging. New motion assessment technologies can evolve into patient-centered eHealth applications on a global scale to support personalized healthcare as well as treatment of disease. However, uncertainty remains on the limits of generalizability of such data, which is relevant specifically for preventive or predictive applications, using normative datasets to screen for incipient disease manifestations or indicators of individual risks.

**Objective:**

This study explored differences between healthy German and Japanese adults in the performance of a short set of six motor tests.

**Methods:**

Six motor tasks related to gait and balance were recorded with a validated 3D camera system. Twenty-five healthy adults from Chiba, Japan, participated in this study and were matched for age, sex, and BMI to a sample of 25 healthy adults from Berlin, Germany. Recordings used the same technical setup and standard instructions and were supervised by the same experienced operator. Differences in motor performance were analyzed using multiple linear regressions models, adjusted for differences in body stature.

**Results:**

From 23 presented parameters, five showed group-related differences after adjustment for height and weight (*R*^2^ between .19 and .46, p<.05). Japanese adults transitioned faster between sitting and standing and used a smaller range of hand motion. In stepping-in-place, cadence was similar in both groups, but Japanese adults showed higher knee movement amplitudes. Body height was identified as relevant confounder (standardized beta >.5) for performance of short comfortable and maximum speed walks. For results of posturography, regression models did not reveal effects of group or body stature.

**Conclusions:**

Our results support the existence of a population-specific bias in motor function patterns in young healthy adults. This needs to be considered when motor function is assessed and used for clinical decisions, especially for personalized predictive and preventive medical purposes. The bias affected only the performance of specific items and parameters and is not fully explained by population-specific ethnic differences in body stature. It may be partially explained as cultural bias related to motor habits. Observed effects were small but are expected to be larger in a non-controlled cross-cultural application of motion assessment technologies with relevance for related algorithms that are being developed and used for data processing. In sum, the interpretation of individual data should be related to appropriate population-specific or even better personalized normative values to yield its full potential and avoid misinterpretation.

**Supplementary Information:**

The online version contains supplementary material available at 10.1007/s13167-021-00236-3.

## Introduction

In recent years, questions on the limitations of current medical practices were raised regarding the individual needs of a patient. Predictive, preventive, and personalized medicine (PPPM) was proposed [1] as a paradigm shift to focus more on the patient as an individual, multi-professional collaboration, and inclusion of new technologies. Currently, there is a lack of literature and research on the implications of quantitative motor function on an individual level, although the predictive and preventive values are frequently discussed.

### Motor function patterns are of value for risk assessment in neurological diseases

Motor function impairment is a hallmark of many neurological disorders, with impact on mobility, functional independence, and well-being of the patient [2]. This is not only important from a clinical but also from a patient’s perspective [3]. Consequently, the observation of motor performance is a relevant diagnostic component, in that abnormal motor functions may predict involvement of specific systems in neurodegenerative disease. For example, higher than normal step variability has been shown to indicate carrier status in spinocerebellar ataxias [4, 5]—even before clinical manifestation—and lower than normal postural stability may predict future decline in gait functions or fall risk in multiple sclerosis [6–9], while a motor-cognitive risk syndrome has been defined as predictive of cognitive decline [10]. It remains to be shown, however, if improvement of such predictive motor features by targeted intervention may also prevent progression events. Further, the observation of motor features has a prominent role in personalized treatment decisions in several neurological disorders. For example, the dosing of therapy—from pharmacotherapy to settings of deep brain stimulation for movement disorders or settings of cerebroabdominal shunts in normal pressure hydrocephalus—is individually tailored to reach an optimum of balance between its beneficial effects on motor functions and immediate side effects or long-term complications of therapy [11]. To date, such decision relies on observation by trained professionals and use of standardized assessments by clinical rating scales to document their findings. This helped to describe effects of intervention or investigate the “natural course” of neurodegenerative diseases and explore their determinants from large scale databases, which, at best, results in valid individual predictors.

### Technology-based objective measures (TOMs) to assess motor function

Instrumented assessment of motor function takes this endeavor even further with the potential to evolve into patient-centered eHealth applications applied on a global scale. Advantages include greater objectivity by reduction of observer bias, yield of inherently quantitative data, expert-independence of assessment, and thus potential for broad and even remote application [12]. In recent years, many technology-based objective measures (TOMs) have been introduced to quantify movement patterns in neurological disorders, ranging from wearable sensors [13–15] to 3D marker-free cameras [16–18]. Many of them may be applied by patients themselves, which would enable access to these TOMs also in resource-poor settings [19]. This development has been noted to shift the need of expertise from data acquisition to the interpretation of data [20, 21]. A convergent line of medical research and technology development may support the interpretation of TOMs, as artificial intelligence approaches aim to effectively integrate multiple and complex data to assist clinical decisions. As an example, automated diagnostic classifiers have been developed from data of a comprehensive instrumental gait assessment battery [22]. Further efforts are made to use such data for predictive and preventive actions [6, 7]. Despite highly active development in this field, independent clinical validations of such approaches are still scarce [23], and their assumptions can be ill-defined [24].

### The question on generalizability of motor patterns

When considering TOMs for data acquisition at a global level, there is not much data available to confirm generalizability of results between culturally diverse populations. In general, the majority of motion analysis research has been conducted in Caucasian subjects and considerably less focused on, e.g., Asians [25–28]. Few cross-cultural analyses [27, 29] stated differences in normative data for motor function tests obtained in Caucasian [30] or in ethnically diverse US American populations, e.g., the NIH toolbox validations [31]. Although differences in test settings may explain inter-site variability to some extent, a specific role of different sociocultural backgrounds on motor habits [32, 33] or valuation of motor patterns [34] as well as differences in body stature [26] have been proposed as alternative or additional explanations for disparities between populations of different ethnic or cultural background.

The objective of this study was to exemplarily identify possible cultural differences in motor function by investigating if and how healthy adult cohorts in Japan and Germany would differ in their motor performance assessed using TOMs. Our study design aimed to carefully exclude effects other than location by group matching, identical set of motor tasks, use of standardized instructions, and supervision by one experienced operator, as well as the same technical setup for recording. The short battery of motor tasks used here was developed for the instrumental assessment of motor function in neurological disorders and previously validated in German samples of healthy subjects, people with multiple sclerosis and Parkinson’s disease [18, 33–35].

## Methods

### Subjects

Two cohorts of healthy volunteers participated in this study (Table 1). The first cohort included 25 healthy young individuals from the physiotherapy and neurology staff of the Chiba University Hospital, Japan, that were recruited in summer 2018. The second cohort consisted of 25 age, sex, and BMI matched healthy volunteers selected from existing study databases at Charité - Universitätsmedizin Berlin, Germany. This matching was achieved by, first, selection of HC between the age of 20 and 40 years from the available 80 datasets of German HC. Second, datasets were confined to those with a body mass index (BMI) between 18 and 29 kg/m^2^ to match the BMI of the Japanese cohort. In this step, German HC subjects were favored with a smaller (than population average) body height and weight to reduce differences to the Japanese cohort. Finally, male and female HC were selected to arrive at a similar sex ratio as the Japanese group. Despite BMI matching being formally achieved, body stature remained statistically different with German controls being taller and heavier (Table 1).

**Table 1:** Descriptive statistics for sample characteristics of Japanese and German cohorts, reported as mean (± standard deviation) and range, as well as respective inferential statistics regarding cohort differences.

	Japanese cohort	German cohort	Cohen’s d	*p*-value^1^
*N*	25	25		
Sex				
F	11	12		.776
M	14	13		
Age [years]	30.3 (±6.2) 23–35	31.5 (±5.09) 22–39	0.211	.458
Height [cm]	166.8 (±8.8) 153–185	173.6 (±9.9) 156–190	0.729	.013
Weight [kg]	60.3 (±9.4) 48–90	68.8 (±9.5) 51–88	0.902	.002
BMI [kg/m^2^]	21.6 (±1.9) 18.9–27.2	22.8 (±2.4) 18.9–28.2	0.562	.053

^1^Calculated with Chi^2^ for sex differences and student’s *t*-test for other parameters

### Motor function assessment

**Fig. 1:**
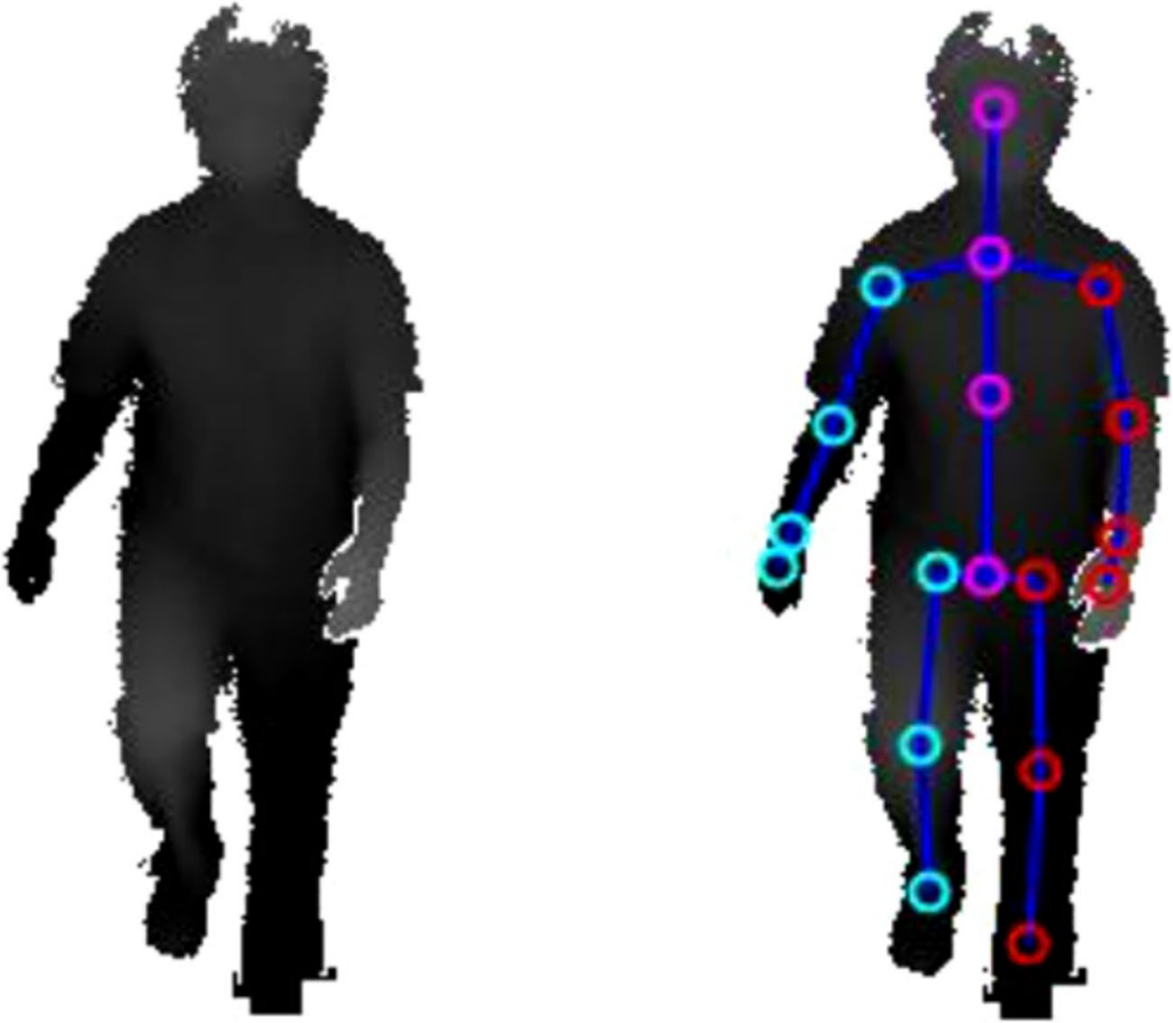
Visualization of recorded depth data (**a**) and placements of artificial anatomical landmarks (**b**)

All motor tasks were captured by an instrumented motion analysis system (Motognosis Labs, Version 1.2.0, Motognosis GmbH, Berlin, Germany) using a real-time 3D consumer camera (Microsoft Kinect V2, Microsoft, Redmond, WA, USA) for motion capture. The recorded movement data included depth data streams (Fig. 1a), 3-dimensional time series of 25 artificial anatomic landmarks (Fig. 1b), and RGB video streams using the Kinect SDK V2.0.1400 (Microsoft, Redmond, WA, USA).

The Perceptive Assessment in Multiple Sclerosis (PASS-MS) protocol was originally developed to analyze motor symptoms in patients with multiple sclerosis (14) (35). It consists of ten different motor tasks to examine upper limb function, gait, and static and dynamic postural control. To focus this study, tasks for the assessment of fine motor control were not included. Therefore, data from the following six tasks were selected (Table 2): short distance gait in self-selected comfortable (SCSW) and maximum speed (SMSW), tandem-gait (SWL), 40-s stepping in place (SIP), standing up and sitting down (SAS), and posturography with open and closed eyes for 20 s each (POCO). For seated assessments, chairs with a backrest, but without armrests, were used. Due to the technical specifications of the sensor, the walk length was limited to about 4m (34). Recording of PASS-MS follows written SOP for setup and test instruction. Identical technical setup and operator training at both study sites were established by one experienced instructor (K.O.).

**Table 2:** Descriptions of the six motor tasks taken from the PASS-MS protocol and derived spatiotemporal parameters

Task name	Short instruction	Movement signals	Derived clinical parameter
Stance with closed feet and open and closed eyes (POCO)	Stand with closed feet and open eyes for 20 s. After audio signal, close the eyes for another 20 s	Body sway (movement of spine base relative to closed feet position)	Average 3D absolute angular sway speed [°/s] and 3D sway deflection range [°] for open and closed phase, as well as their ratios between open eye to closed eye phase of measurement (Romberg ratio)
Short comfortable speed walk (SCSW)	After audio signal, walk directly towards the sensor at comfortable speed	Spatial body movement (spine base movement) and ankle movements in walk direction	Average gait speed [m/s], cadence [steps/min], and average step length [cm]
Short maximum speed walk (SMSW)	After audio signal, walk directly towards the sensor at maximum speed	Spatial body movement (spine base movement)	Average gait speed [m/s]
Short line walk (SLW)	After audio signal, walk on an imaginary line directly towards the sensor with heel touching the toes	Spatial body movement (spine base movement) ML trunk deflection (movement of shoulder center relative to spine base)	Average progression speed [m/s], standard deviation of angular trunk sway in ML direction [°], and standard deviation of angular arm movements in V direction [°]
Stand up and sit down (SAS)	After audio signal, stand up and wait for second audio signal, then sit down	Trunk deviation (movement of shoulder center relative to spine base), hand range of motion in AP direction	Time needed for standing up and sitting down [s], deviation of trunk during transitions [cm] in AP, and range of motion of hands in AP direction [cm]
Stepping in place (SIP)	Walk on the spot at comfortable pace for 40 s	AP-V displacement of the knees	Average knee amplitude [cm], amplitude asymmetry [%], arrhythmicity [%], and cadence [steps/min] [35]

*ML* medio-lateral, *AP* anterior-posterior, *V* vertical

Subjects were either placed in 2.5m (SIP, SAS, POCO) or in 5m distance (SCSW, SMSW, SLW) to the camera system in a standing or sitting position as specified for each test. Two immediate measurement repetitions were performed for SAS, SCSW, SMSW, and SLW tasks, resulting in three recordings per patient per assessment for these tasks. Recorded data were used to extract 23 spatiotemporal parameters that describe different aspects of motor performance. For SAS, SCSW, SMSW, and SLW, spatiotemporal parameters were reported as an average of all three repetitions.

### Statistical analysis

Normality of parameters was explored by inspection of group-wise histograms and by Shapiro-Wilk tests. The Shapiro-Wilk tests showed normal distribution of 13 parameters in Japanese and 18 parameters in German cohort. The non-normal distribution of short comfortable gait parameters in the Japanese cohort was mainly explained by two data points of participants who featured high gait speed and large step length. Mean, standard deviation (SD), and coefficient of variation (CV) were computed for all derived parameters for each group. Independent *t*-test was performed to compare measurements at group level where effect size of differences was reported as Cohen’s d. Additionally, to account for remaining differences in stature between groups, multiple linear regression models with height and weight as fixed effects were used. Model significance was assumed when *p*<.05. Statistical testing was not corrected for multiple comparisons due to the exploratory nature of this work. Calculations were performed using Python 3.5, the scipy-package version 0.18.1 and the statsmodels-package version 0.10.1. Diagrams were created with seaborn-package version 0.7.1 and Matplotlib-package version 2.0.0.

## Results

Descriptive statistics of all 23 parameters for both cohorts are given in Table 3. Within-group variance per parameter, as indicated by CV, was of similar magnitude in both groups (range 0.06 to 0.90). The highest CV values were found in measurements of knee amplitude asymmetry while stepping in place (Japanese: .86; German: .90), variability of trunk sway (Japanese: .70; German: .47), and arm movement variability in short line walk (Japanese: .66; German: .65).

**Table 3:** Descriptive statistics of 23 spatiotemporal parameters derived from six different motor tasks, presented for German (*N*=25) and Japanese (*N*=25) cohort

		Japanese mean (SD)	Japanese CV	German mean (SD)	German CV	*t*-test *p*-value	Cohen’s d
Stance with closed feet (POCO)	3D deflection range (open eyes) [°]	1.01 (0.55)	0.54	0.80 (0.41)	0.50	.135	−0.43
	3D sway speed (open eyes) [°/s]	0.22 (0.08)	0.39	0.19 (0.05)	0.27	.297	−0.30
	3D deflection range (closed eyes) [°]	1.10 (0.41)	0.37	1.03 (0.56)	0.53	.609	−0.15
	3D sway speed (closed eyes) [°/s]	0.31 (0.09)	0.29	0.26 (0.10)	0.37	.065	−0.53
	Romberg ratio of 3D deflection range	1.32 (0.65)	0.49	1.43 (0.74)	0.51	.563	0.16
	Romberg ratio of 3D sway speed	1.55 (0.48)	0.31	1.37 (0.46)	0.33	.199	−0.37
Short comf. speed walk (SCSW)	Gait speed [m/s]	1.16 (0.14)	0.12	1.16 (0.18)	0.16	.923	−0.03
	Step length [cm]	67.19 (7.86)	0.12	69.41 (8.06)	0.12	.327	0.28
	Cadence [steps/min]	116.1 (7.3)	0.06	110.8 (10.7)	0.10	**.049**	-0.57
Short max. speed walk (SMSW)	Gait speed [m/s]	1.79 (0.18)	0.10	1.73 (0.16)	0.09	.185	−0.38
Short line walk (SLW)	Progression speed [m/s]	0.37 (0.09)	0.24	0.34 (0.07)	0.19	.164	−0.40
	Variability of angular trunk sway (ML) [°]	1.32 (0.92)	0.70	1.94 (1.0)	0.47	**.026**	0.65
	Variability of arm movements [°]	3.44 (2.26)	0.66	5.64 (3.67)	0.65	**.015**	0.71
Stand up and sit down (SAS)	Stand up transition time [s]	1.34 (0.19)	0.14	1.50 (0.18)	0.12	**.004**	0.86
	Stand up trunk deflection (AP) [cm]	14.2 (2.84)	0.20	14.7 (2.80)	0.19	.608	0.15
	Stand up hand deflection (AP) [cm]	7.24 (3.0)	0.42	11.7 (4.3)	0.37	**.000**	1.19
	Sitting down transition time [s]	1.36 (0.23)	0.16	1.61 (0.25)	0.15	**.001**	1.03
	Sitting down trunk deflection (AP) [cm]	11.8 (2.58)	0.22	14.6 (2.98)	0.20	**.001**	1.01
	Sitting down hand deflection (AP) [cm]	5.6 (2.43)	0.43	10.2 (2.88)	0.28	**.000**	1.72
Stepping in place (SIP)	Cadence [steps/min]	108.8 (14.3)	0.13	104.3 (19.0)	0.18	.351	−0.27
	Knee amplitude (AP) [cm]	23.6 (5.13)	0.22	19.8 (6.53)	0.33	**.025**	−0.65
	Amplitude asymmetry [%]	6.73 (5.77)	0.86	10.78 (9.72)	0.90	**.**079	0.51
	Arrhythmicity [%]	8.97 (3.34)	0.37	8.58 (4.29)	0.50	.719	−0.10

*p* <0.05 marked in bold

*SD* standard deviation, *CV* coefficient of variation, *AP* anterior-posterior, *ML* medio-lateral

**Fig. 2:**
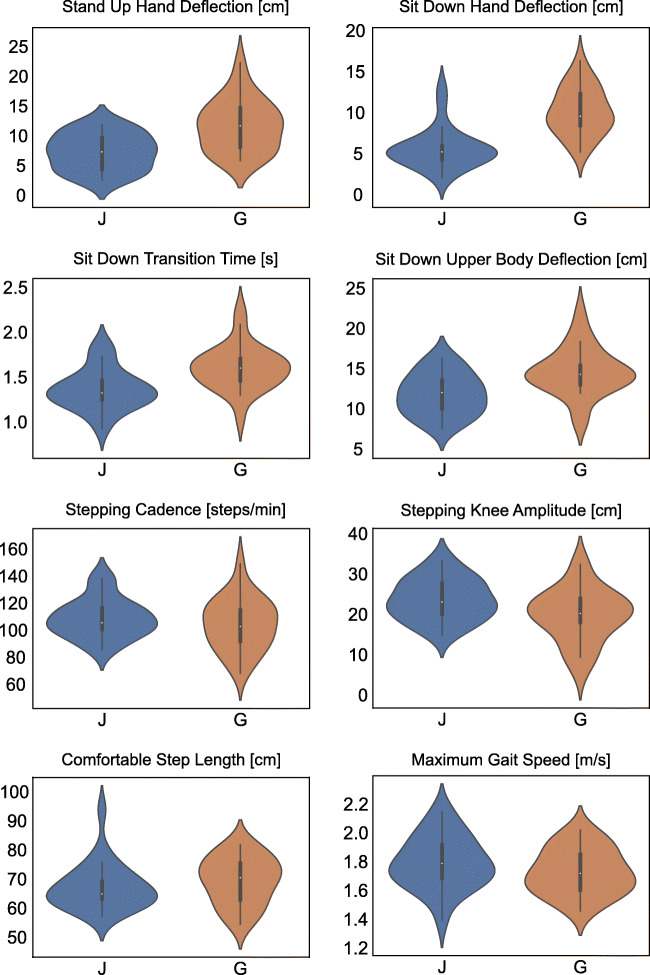
Violin plots of eight spatiotemporal parameters that showed statistical significant differences (*p* < .05) in independent *t* test between Japanese (J) and German (G) cohorts

Although groups were matched for age, sex, and BMI, the cohorts of Japanese and German healthy adults differed in unadjusted comparison for 9 of our 23 parameters, all with Cohen’s d >0.5 (Table 3). Largest differences were found for transition times and hand movements during stand up and sit down (d>.85). Further differences were seen in knee amplitudes of stepping in place, cadence in short comfortable speed walk, and trunk sway and arm movement variability during short line walk (see also Fig. 2).

In order to correct for remaining differences in body stature between cohorts, multiple linear regression models were used. In these models, significant group effects remained only for parameters of SAS and SIP task performance (Table 4). In SAS, the Japanese cohort showed overall less use of arms when standing up from sitting position and faster transition times and smaller ranges of trunk and arm motion, for the transition to sitting. Trunk range of motion in both transitions and hand use during stand up were additionally influenced by body height (*p*-value<.05). In SIP, the German cohort featured smaller knee amplitudes. A visualization of a typical performance from each group is provided for these tasks as de-personalized video material in the supplementary material 1.

**Table 4:** Multiple linear regression models to analyze differences between Japanese and German young healthy adults (group effect) with correction for individuals’ body height and weigh

		Overall		Group			Height			Weight		
		F test *p*-value	*R*^2^	*p*-value	Std. beta.	CI	*p*-value	Std. beta	CI	p-value	Std. beta	CI
Stance with closed feet (POCO)	3D deflection range (open eyes) [°]	0.248	0.085	0.210	0.20	(−0.12; 0.51)	0.170	−0.31	(−0.76; 0.14)	0.346	0.22	(−0.24; 0.68)
	3D sway speed (open eyes) [°/s]	0.350	0.068	0.290	0.17	(−0.15; 0.48)	0.157	−0.32	(−0.77; 0.13)	0.185	0.31	(−0.15; 0.78)
	3D deflection range (closed eyes) [°]	0.913	0.011	0.787	0.04	(−0.28; 0.37)	0.755	−0.07	(−0.54; 0.39)	0.962	−0.01	(−0.49; 0.47)
	3D sway speed (closed eyes) [°/s]	0.139	0.112	0.056	0.30	(−0.01; 0.61)	0.202	−0.28	(−0.72; 0.16)	0.156	0.33	(−0.13; 0.78)
	Romberg ratio of 3D deflection range	0.949	0.008	0.637	-0.08	(−0.40; 0.25)	0.879	0.04	(−0.43; 0.50)	0.955	−0.01	(−0.49; 0.47)
	Romberg ratio of 3D sway speed	0.560	0.043	0.164	0.22	(−0.09; 0.54)	0.828	−0.05	(−0.51; 0.41)	0.560	0.14	(−0.33; 0.61)
Short comf. speed walk (SCSW)	Gait speed [m/s]	0.119	0.118	0.994	0.00	(−0.31; 0.31)	**0.019**	0.53	(0.09; 0.97)	**0.041**	−0.47	(−0.93; −0.02)
	Step length [cm]	**0.001**	0.296	0.884	-0.02	(−0.29; 0.25)	**<.001**	0.76	(0.36; 1.15)	0.099	−0.34	(−0.74; 0.07)
	Cadence [steps/min]	**0.011**	0.211	0.446	0.11	(−0.18; 0.40)	0.907	−0.02	(−0.44; 0.39)	0.079	−0.38	(−0.81; 0.05)
Short max. speed walk (SMSW)	Gait speed [m/s]	**0.014**	0.203	0.061	0.28	(−0.01; 0.57)	**0.006**	0.60	(0.18; 1.02)	0.182	−0.29	(−0.72; 0.14)
Short line walk (SLW)	Progression speed [m/s]	0.582	0.041	0.219	0.20	(−0.12; 0.52)	0.805	−0.06	(−0.51; 0.40)	0.854	0.04	(−0.43; 0.52)
	Variability of angular trunk sway (ML) [°]	0.073	0.139	0.056	-0.30	(−0.60; 0.01)	0.172	−0.30	(−0.73; 0.13)	0.191	0.30	(−0.15; 0.74)
	Variability of arm movements [°]	**0.017**	0.196	0.050	−0.29	(−0.59; −0.00)	0.065	−0.39	(−0.81; 0.03)	**0.045**	0.44	(0.01; 0.88)
Stand up and sit down (SAS)	Stand up transition time [s]	**0.002**	0.273	0.068	−0.26	(−0.54; 0.02)	0.219	0.25	(-0.15; 0.65)	0.507	0.14	(−0.27; 0.55)
	Stand up trunk deflection (AP) [cm]	**0.018**	0.194	0.672	0.06	(−0.23; 0.36)	**0.012**	0.55	(0.13; 0.97)	0.547	−0.13	(−0.56; 0.30)
	Stand up hand deflection (AP) [cm]	**<.001**	0.363	**0.001**	−0.47	(−0.73; −0.21)	**0.016**	0.46	(0.09; 0.84)	0.177	−0.26	(−0.65; 0.12)
	Sitting down transition time [s]	**0.005**	0.243	**0.007**	−0.40	(−0.69; −0.12)	0.388	0.18	(−0.23; 0.58)	0.999	0.00	(−0.42; 0.42)
	Sitting down trunk deflection (AP) [cm]	**0.001**	0.285	**0.004**	−0.41	(−0.69; −0.14)	**0.040**	0.42	(0.02; 0.81)	0.253	−0.23	(−0.64; 0.17)
	Sitting down hand deflection (AP) [cm]	**<.001**	0.460	**<.001**	−0.65	(−0.89; −0.41)	0.151	0.25	(−0.09; 0.59)	0.278	−0.19	(−0.55; 0.16)
Stepping in place (SIP)	Cadence [steps/min]	0.074	0.138	0.866	−0.03	(−0.33; 0.28)	0.933	−0.02	(−0.45; 0.42)	0.105	−0.37	(−0.82; 0.08)
	Knee amplitude (AP) [cm]	**0.033**	0.171	**0.012**	0.39	(0.09; 0.68)	0.087	0.37	(−0.06; 0.80)	0.514	−0.14	(−0.58; 0.30)
	Amplitude asymmetry [%]	0.386	0.063	0.104	−0.26	(−0.58; 0.06)	0.997	0.00	(−0.45; 0.45)	0.918	−0.02	(−0.49; 0.44)
	Arrhythmicity [%]	0.678	0.032	0.936	0.01	(−0.31; 0.33)	0.285	−0.25	(−0.71; 0.21)	0.637	0.11	(−0.36; 0.59)

Reported beta coefficients are standardized; p<.05 marked bold

*CI* Confidence Interval

Difference in measures of cadence during short comfortable speed walk disappeared after correction for body height and weight. For step length and gait speed during short comfortable speed walk as well as gait speed during short maximum speed walk, results suggested an influence of the individual’s body height, but no independent effect of group was seen.

A weak effect of body weight was seen for the comfortable gait speed and variability of arm movement during tandem walk, a parameter thought to indicate active counterbalance. For static stance with open and closed eyes, a test of postural control, no effect of group, and no influence of body height or weight were observed.

It should be noted that rather small amounts of variance were explained by each linear model, with the highest *R*^2^ in hand deflection during SAS (up: R^2^=.36; down: R^2^=.46, both p<0.001). Comparison of standardized beta coefficients showed similar magnitudes of influences by group and body height in stand up as well as sit down hand deflection range.

## Discussion

In this study, motor performances of young healthy adults from Germany and Japan were explored during six motor tests which are frequently used for the quantitative assessment of gait and balance functions. While the amount of sway in quiet standing was expectedly low in both cohorts and unaffected by individuals’ height and weight, performances in the other five tasks showed group-related differences (stand up and sit down, stepping in place) or influences of body weight (tandem walk) or body height (short comfortable and maximum speed walk).

### Matching of different populations—inevitable bias of ethnic differences in body stature

Possible sources of variance were carefully considered and minimized by study design in order to isolate a possible effect of the study site. Data acquisition in both sites used consistent technical setup, standard test instructions, and assessment procedures. The operators were trained by the same instructor, who also supervised measurement recordings at both sites. Further, cohorts were matched for age, sex, and BMI.

Cohorts were confined to young adult age only (20–40 years old), as aging is a well-known factor for various motor outcomes [30, 36]. This way, age was excluded as a confounder for between-group comparisons, and no relevant differences would be expected in leg strength or general physical capacity in this age group. With respect to body stature, despite efforts of matching for BMI, population differences in body height and weight could not be fully eliminated due to ethnic differences. The body mass index in Asian populations has a lower normative range compared to populations of western origin [37]. To compensate for this, the German sample was selected to represent a similar body type. Hence, German subjects with smaller than national average BMI and height were favored. Still, full matching was not feasible, and a systematic bias remained with taller stature in German subjects that may contribute to between-group differences.

In fact, stature-related influences on motor performance were seen for several parameters, specifically for spatial parameters of gait function. The slightly shorter step length in Japanese individuals in the test of short walk at comfortable speed was explained by group differences in body height alone. This is in line with the known relation of step length to body height [38], which seems to apply to German and Japanese subjects equally. Similarly, an effect of height was also seen for gait speed in both measurement conditions. Generally, the gait speed observed in our study was consistent with previous data on short comfortable and maximum speed walks [31, 36] published from European cohorts. Existing reports of cultural differences in gait behavior [26, 29] did not compensate for (unreported) differences in stature and body height which limits comparability but would explain their findings.

However, even after correction for differences in body stature, Japanese and German healthy adults differed in performance of stand up and sit down as well as stepping in place task.

### Differences not explained by difference in body stature

In stand up and sit down task, Japanese subjects, at group level, featured much faster performance and less antero-posterior trunk deflection for stand-to-sit transitions and generally much less use of arms during transitions compared to German subjects. The standardized beta coefficients showed influences of similar magnitude for group and body height on these arm movements. Interestingly, group differences in stand-to-sit transition time was not explained by stature bias. Transitions from or to sitting positions are some of the most common motor tests performed in geriatric screening (e.g., as part of the timed up and go [39] or as sit-to-stand test [40]) and are considered to reflect different aspects of physical functions such as leg strength, postural control, and general physical fitness. The most commonly used read-out is the time needed for the sit-to-stand transition (stand up time) [41], for which only few normative data are available for younger age groups. Performance times in a small UK sample (n = 15, mean age 26 years ± 6 years SD) [42] were 1.43s and thus very similar to our observations, while somewhat slower performance (2.42s) was reported from a small Italian cohort (n=13, mean age 35 years ± 5 years SD) [43] which may be explained by differences in age, instruction bias, cultural bias, or even chance, given the small sample sizes.

When analyzing stepping in place behavior, groups differed in knee movement amplitudes of stepping, where the German cohort performed smaller movements in comparison to the Japanese cohort while maintaining comparable cadence. While the Japanese cohort consistently featured amplitudes above 15cm, 20% of German subjects showed amplitudes smaller than 15cm. As the assessment of stepping in place is rather used in persons with Parkinson’s disease with time-based measures as the typical read-out, no normative data or reference exist for this parameter. Own results in a small (German) cohort of people with Parkinson’s disease report even lower knee amplitudes in this test (mean: 12.5cm, standard deviation: 7.4 cm ), indicative of hypokinesia [35].

### Cultural bias as a possible explanation

Since the test setting was supervised by the same experienced instructor (K.O.) at both sites, we consider the observed differences in performance to be an expression of cultural bias which could be retrospectively explained by different sociocultural backgrounds. In Japanese culture, standing up is often executed as common courtesy without much reluctance, i.e., when a higher ranked person is entering a room. This is usually not the case in western society and not practiced in Germany. Therefore, Japanese participants may have adopted a different motor habit specifically in execution of this task and not in others such as the short or line walks. The more marching-like behavior observed in Japanese adults while stepping in place might be explained by mandatory sport and marching training in Japanese schools which is not part of educational practice in Germany.

With respect to a possible relevance of cultural bias for distinct motor tasks and spatiotemporal parameters, the amount of variance explained (*R*^2^) was generally small in our models. However, an independent effect of group was demonstrated even in these comparatively small cohorts, which reflects an overt performance difference that is easily observed (see supplemental video material).

### Importance of standardized task instruction

Although confounding effects were either tried to be minimized by study design or compensated for during statistical testing, nonlinear effects might still be present. For example, standard instruction of motor tasks had to be translated from German to English to Japanese in cooperation with trained Japanese health professionals, but without standard cross-cultural validation [44], which may have added variance in connotations. As possible alternative explanations, the role of individual motor strategies [45] or effects of attention or fatigue on motor performance [46] might also be considered in a further study. As outlined in many reviews on quantitative motor outcomes [27], standardized reporting of test setting, test instructions, and sample characteristics is crucial for the proper interpretation of test results, specifically in the application of normative values. This is most relevant when TOMs are used to screen for predictive motor features or manifestation of disease or to demonstrate effectiveness of preventive medicine.

### Implication of our findings for PPPM

Since one of the main aspects of PPPM is to provide personalized medical services, the understanding and modelling of a patient’s situation is an essential aspect [47]. This should include the awareness about systematic bias in results of diagnostic observation or investigation. The existence of the presented biases in motor function implies that population-specific normative datasets should be preferred as reference for the individual interpretation of technology-based objective measures.

Interestingly, this need has not been addressed in recent publications of large normative datasets [31, 48]. With the availability of individual normative reference values, e.g., by population-specific databases, by transformation to confounder-independent variables, or even use of previous data acquisitions as personalized reference in follow-up investigations, motor function may become more common as screening or monitoring tool. The presented similarities, i.e., lack of such biases, in motor function patterns between Japanese and German young adults may indicate higher individual variability outweighs population-specific differences.

## Summary and conclusion

With this study, we aim to increase the awareness of potential cultural and/or ethnic biases in the instrumental assessment of motor functions. In the context of an increasingly global health perspective in research, such biases need also be considered in multi-national data acquisition and analysis. Specifically for the use of such data to train machine learning models, unrecognized biases may increase the error rates or decrease generalizability [49, 50]. In conclusion, new technologies for personalized monitoring of motor function are promising in many regards, but they should be analyzed regarding underlying confounding effects including culture or ethnicity to prevent biased results.

## Supplementary Information

ESM 1 (AVI 4693 kb)

ESM 2 (AVI 4369 kb)

## Data Availability

Anonymized spatiotemporal parameters may be provided upon reasonable request.

## Code availability

Code for statistical and data analysis will be provided upon request.
